# Higher Area Deprivation Index scores predict incomplete pre-operative high-resolution manometry in Achalasia patients undergoing peroral endoscopic myotomy

**DOI:** 10.1007/s00464-026-13107-7

**Published:** 2026-07-09

**Authors:** Yassmin Hegazy, Mary Michael Shannon, Dylan Mcclain, Justin Labonte, Venkata Seerapu, Eldrin Bhanat, Salit Chakma, Fazlay Faruque, Jacob Moremen

**Affiliations:** 1 Division of Gastroenterology, Department of Medicine, University of Mississippi, Jackson, MS USA; 2 School of Medicine, University of Mississippi, Jackson, MS USA; 3 Department of Surgery, University of Mississippi, Jackson, MS USA; 4 Geospatial Health Research Lab, John D. Bower School of Population Health (SOPH), University of Mississippi, Jackson, MS USA; 5 Division of Thoracic Surgery, Department of Surgery, University of Mississippi, Jackson, MS USA

**Keywords:** Achalasia, Area Deprivation Index, Peroral endoscopic myotomy, High-resolution manometry, Social determinants of health, Healthcare disparities

## Abstract

**Background:**

The Area Deprivation Index (ADI) is an important measure of neighborhood-level socioeconomic status (SES). Our study evaluates the correlation of ADI scores to post-operative clinic follow up compliance and completion of pre-operative high-resolution manometry (HRM) in patients with Achalasia undergoing Peroral Endoscopic Myotomy (POEM).

**Methods:**

We conducted a retrospective cohort study of adult patients undergoing POEM at a single tertiary care center between March 2017 and December 2024 (N = 193). ADI was assigned using 2023 national rankings based on patient residential addresses. Analyses were restricted to patients with available ADI data (n = 181). Multivariable logistic regression was performed adjusting for age, travel distance, sex, body mass index (BMI), and smoking status.

**Results:**

Patients who did not complete pre-operative HRM had higher ADI scores (89 [IQR 21]) compared to those who did (85 [IQR 29]), (p = 0.015). Patients who did not attend initial or overall post-operative follow-up also had higher ADI scores compared to those who completed follow-up. Median ADI was higher among patients with incomplete initial follow-up (90 [IQR 17] vs 85 [IQR 28]; p = 0.068) and overall follow-up (91 [IQR 18] vs 85 [IQR 27]; p = 0.064); however, these differences did not reach statistical significance on non-parametric testing. Mean values demonstrated similar patterns but were statistically significant on parametric analysis (p = 0.010 and p = 0.013, respectively).

In multivariable logistic regression analysis, higher ADI rank was independently associated with increased odds of failing to complete pre-operative HRM (OR 1.04, 95% CI 1.01–1.07, p = 0.005). Age, travel distance, sex, BMI, and smoking status were not significantly associated with HRM completion (all p > 0.05).

**Conclusion:**

Patients with higher ADI scores were less likely to complete HRM pre-operative testing. ADI scores can be used as a measure to risk stratify Achalasia patients undergoing POEM.

**Graphical abstract:**

**Figure:**
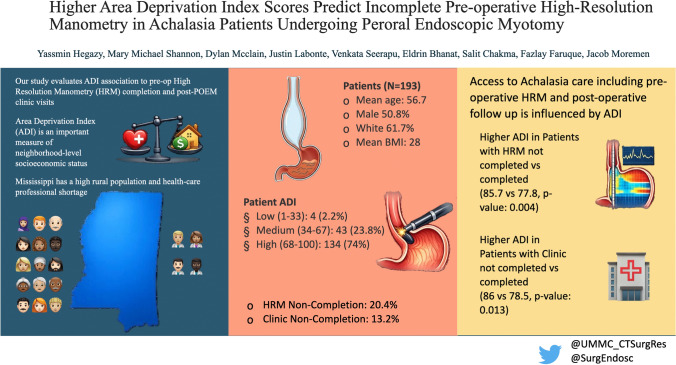

Achalasia is an esophageal motility disorder characterized by impaired lower esophageal sphincter relaxation causing high symptom burden including progressive dysphagia, regurgitation, chest pain, and weight-loss [1]. High-Resolution Manometry (HRM) is the gold standard for diagnosis of Achalasia using the Chicago 4 Classification, characterized by esophageal aperistalsis and integrated relaxation pressure (IRP) greater than 15 mmHg [2]. Peroral endoscopic myotomy (POEM) has become an established minimally invasive treatment across all Achalasia subtypes [3, 4]. Multiple studies have shown high clinical success and symptomatic improvement compared to other treatments such as Pneumatic Dilations and Laparoscopic Heller Myotomy, positioning POEM as a first line therapy for treatment of Achalasia [5–7].

While HRM aids in confirming diagnosis and guiding the treatment plan for patients with Achalasia, HRM requires advanced equipment, specialized provider interpretation, and often referral to tertiary motility centers that can create barriers for patients in socioeconomically disadvantaged communities [8, 9]. The Area Deprivation Index (ADI) measures neighborhood level social determinants of health including income, education, housing, and transportation using national census data that quantifies neighborhood disadvantage [10, 11]. ADI has been used as a metric for targeting healthcare inequity with prior studies indicating that high ADI scores reflective of increased deprivation were consistently linked with chronic disease burden, poor surgical outcomes, and reduced utilization of recommended healthcare resources [12–14].

Mississippi has consistently ranked on the lower end of national measures for healthcare access and outcomes. Approximately 54.4% of Mississippi’s population resides in rural areas, compared to the national average of 20.4% and 91% of Mississippi’s counties are designated as Medically Underserved Areas (MUA) [15–17]. Additionally, 58 of the state’s 82 counties have Health Professional Shortage Area (HPSA) scores of 20 or greater, with a score of 26 indicative of the most severe shortage across dental health, primary care, and mental health services [18]. These workforce deficits and large rural population may contribute to limited access to healthcare, reflective of the state’s socioeconomic disadvantages relative to national standards. Prior multidisciplinary interventions targeting disadvantaged communities have demonstrated improvements in the state’s population health, highlighting the potential impact of identifying and focusing intervention in an effort to reduce inequities [19, 20]. Rodriguez et al. examined disparities in access to endoscopy for patients with upper gastrointestinal bleeding and found that historically marginalized racial groups and patients receiving rural care had lower odds of undergoing upper endoscopy [21]. In contrast, Stuyak et al. reported that although barriers to access exist at high-volume medical centers, rural patients experienced comparable short-term surgical outcomes and were more likely to receive direct surgical referrals [22].

While socioeconomic disparities relating to healthcare are well-documented, there is limited evidence on its impact on specialized pre-operative testing including HRM or compliance with post-operative follow up in patients with Achalasia undergoing Peroral Endoscopic Myotomy (POEM). While ADI has been used in risk stratifying patients with chronic illnesses and healthcare access related to surgical procedures, our study specifically examines ADI application to Achalasia care in patients undergoing POEM including pre-operative HRM completion and post-operative clinic follow up adherence. Our study hypothesizes that higher ADI is associated with lower rates of HRM completion and reduced rates of post-operative follow up in our patient population.

## Materials and methods

Our study was a retrospective cohort study evaluating patients with Achalasia undergoing POEM at our institution between March 2017 and December 2024. Institutional Review Board approval was obtained with waiver of informed consent (UMMC IRB 2025-4). Inclusion criteria included patients age 18 years or older, patients who underwent POEM for treatment of Achalasia, and those with a residential ZIP code. Exclusion criteria included patients without a home address, with invalid or incomplete address that were not geocoded, with non-Mississippi address, or with suppressed ADI scores. Suppressed ADI scores included group quarters (greater than 33.3% of housing units), population less than 100, and/or fewer than 30 housing units.

**Fig. 1:**
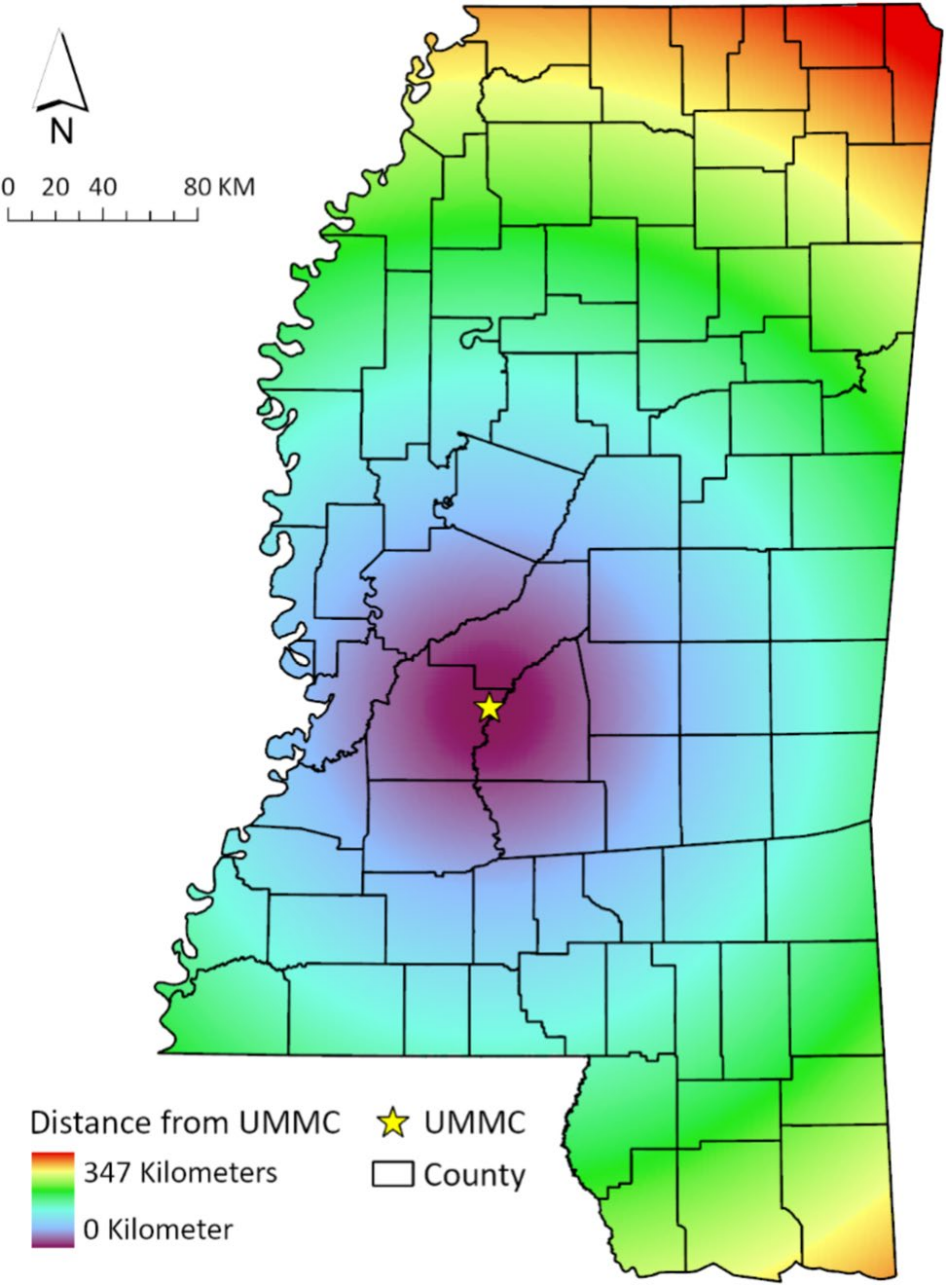
Distance from the University of Mississippi Medical Center (UMMC) for any given location of Mississippi

**Fig. 2:**
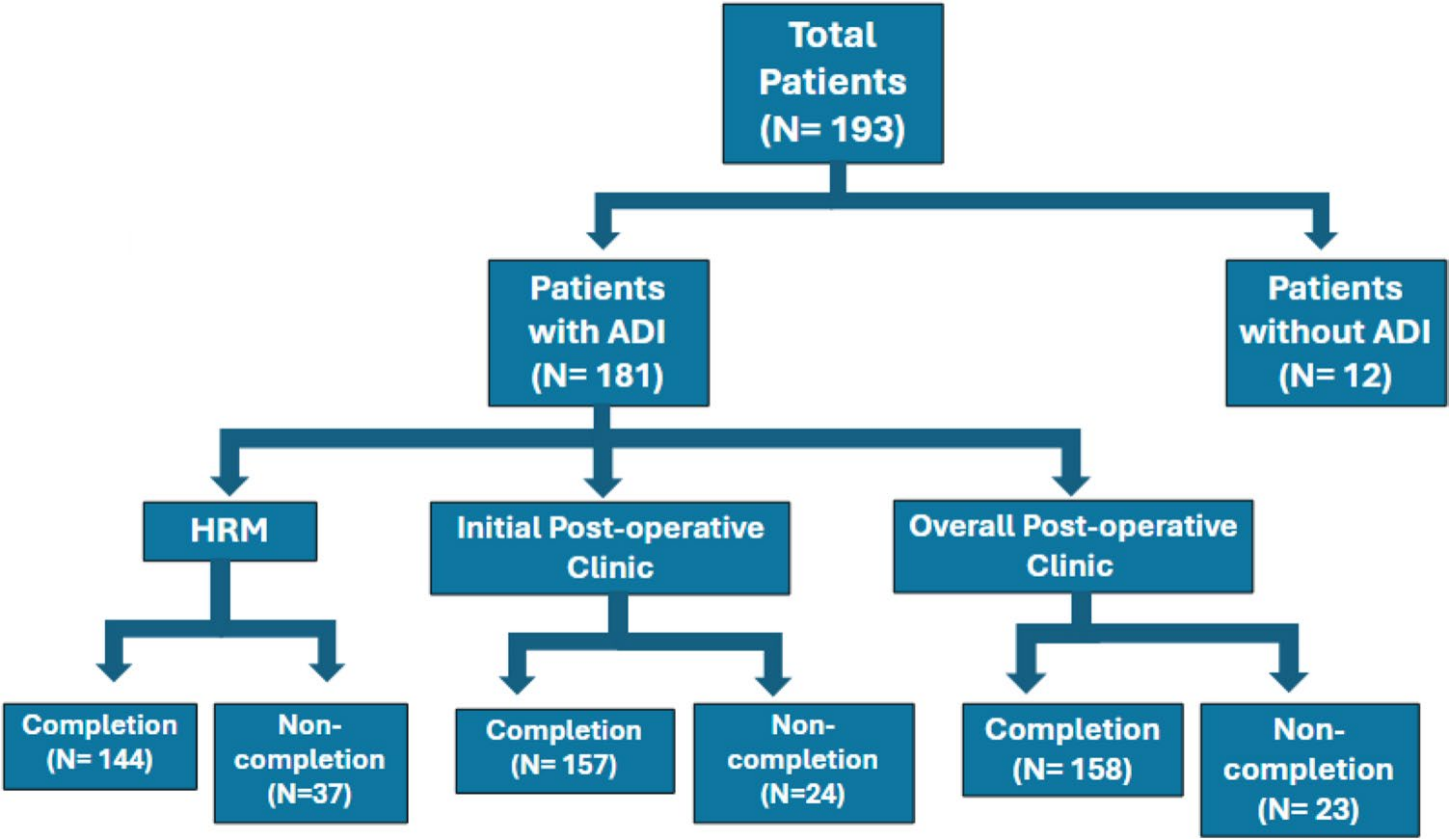
Patient study cohort

ADI national rank scores of 2023, as block groups using patient home addresses, was used to calculate neighborhood socioeconomic status (SES) with a value of 1 indicating the least deprivation and higher values representing the most disadvantaged neighborhoods [23]. The addresses were geocoded to their exact locations, and block group-level ADI scores were extracted using these point locations. National ADI was evaluated using two predefined groups including continuous ADI which was based on 1–100 scale and three grouped categories including low deprivation (1–33) moderate deprivation (34–67), and high deprivation (68–100). Patients’ home addresses were used to determine their distances from the University of Mississippi Medical Center (UMMC) and Fig. 1 shows a generalized illustration of distance from UMMC for the entire state. Incomplete HRM includes any patient unable to complete the study for any reason (tolerability, cost, transportation, etc.). Initial follow up is the first visit within 3 months of the procedure, while overall follow up indicates any return to clinic from the time of the procedure to the end of study period (Fig. 2).

Multivariable logistic regression models were constructed to evaluate factors associated with pre-operative HRM completion and post-operative follow-up. The dependent variable for the HRM model was coded such that HRM non-completion was treated as the event (No = 2, Yes = 1), and therefore odds ratios represent the likelihood of failing to complete HRM. Covariates included age, travel distance, sex, body mass index (BMI), and smoking status, selected based on clinical relevance and consideration of the limited number of outcome events to reduce the risk of overfitting. Travel time was initially considered but excluded from final models due to significant collinearity with travel distance (variance inflation factor > 80), to improve model stability. Analyses involving ADI were restricted to patients with available ADI data (n = 181). Model calibration was assessed using the Hosmer–Lemeshow goodness-of-fit test, and discrimination was evaluated using receiver operating characteristic (ROC) curves with area under the curve (AUC). Additional variables such as race, insurance status, and referral source were considered but were not included in the final models due to limited event counts and incomplete data availability. Statistical significance was defined as a two-sided p-value < 0.05. All analyses were performed using SPSS.

## Results

Our cohort (N = 193) had a mean age of 56.74 ± 18.23, 50.8% were male with a mean BMI of 28.06 ± 8.13. 40.9% had a smoking use history, and 17.6% had a psychiatric co-morbidity (Table 1). ADI score was assessable in 181 patients and analyzed as continuous variables (1–100) and categorized into predefined ADI groups (ADI 1–33, 34–67, and 68–100). Continuous predictors were modeled on their natural scale. Odds ratios for ADI represent the effect per 1-point increase on the 1–100 scale, and travel distance represents the effect per 1-mile increase. Analyses involving ADI were restricted to this subset. ADI was unavailable for 12 patients due to incomplete or non-geocodable address information.

**Table 1:** Achalasia patient demographics undergoing POEM

Demographic	Number of patients N (%)
Sex	
Male	98 (50.8)
Female	95 (49.2)
Race	
White	119 (61.7)
African-American	72 (37.3)
Other	2 (1)
Smoking use	
None	113 (58.9)
Prior smoker	44 (22.9)
Current smoker	35 (18.2)
Past medical history	
Hypertension	94 (59.5)
Diabetes mellitus	34 (21.5)
Congestive heart failure	8 (5.1)
Coronary artery disease	27 (17.1)
Asthma	14 (8.9)
Chronic obstructive pulmonary disease	12 (7.6)
Obstructive sleep apnea	16 (10.1)
Gastroesophageal reflux disease	76 (48.1)
Psychiatric disease*	34 (21.5)
Past surgical history	
Heller myotomy	13 (12.3)
Fundoplication	8 (7.5)
Esophageal dilation	78 (73.6)
Botox	44 (41.5)
ASA score	
1	2 (1.1)
2	91 (47.9)
3	90 (47.4)
4	7 (3.7)
ADI score (N = 181)	
Low (1–33)	4 (2.2)
Medium (34–67)	43 (23.8)
High (68–100)	134 (74)

*CHF* congestive heart failure, *CAD* coronary artery disease, *COPD* chronic obstructive pulmonary disease, *OSA* obstructive sleep apnea, *GERD* gastroesophageal reflux disease, *ASA* American Society of Anesthesiologists

*Psychiatric disease includes depression, anxiety, and bipolar disorder

Overall, among patients with available ADI data (n = 181), 37 (20.4%) did not complete pre-operative HRM, and 23 (12.7%) did not attend any post-operative follow-up. Patients who did not complete pre-operative HRM had higher ADI scores compared to those who completed manometry. Median ADI was higher among patients who did not complete HRM compared to those who did (89 [IQR 21] vs 85 [IQR 29]), and this difference was statistically significant on non-parametric analysis (Mann–Whitney U, p = 0.015). Mean ADI values demonstrated a similar pattern (85.73 ± 13.51 vs 77.80 ± 17.80; p = 0.004).

Patients who never attended a follow up had higher ADI scores compared to those who completed follow up. Median ADI was 91 [IQR 18] among patients with incomplete follow up versus 85 [IQR 27] among those who completed follow up; however, this difference did not reach statistical significance (Mann–Whitney U, p = 0.064). Mean values showed a similar trend (86.00 ± 12.07 vs 78.46 ± 17.79; p = 0.013).

A similar pattern was observed for initial post-operative follow up, where patients who did not attend follow up had higher ADI scores (median 90 [IQR 17] vs 85 [IQR 28]); however, this difference did not reach statistical significance (p = 0.068).

Adjusting for age, travel distance, sex, BMI, and smoking status, multivariable logistic regression analysis demonstrated that higher ADI national rank was independently associated with increased odds of failing to complete pre-operative HRM (OR 1.04, 95% CI 1.01–1.07, p = 0.005), interpreted per 1-point increase in ADI. Travel distance (OR 1.00, 95% CI 0.99–1.00, p = 0.335), age (OR 1.00, 95% CI 0.98–1.02, p = 0.775), sex (OR 1.19, 95% CI 0.55–2.60, p = 0.654), BMI (OR 0.98, 95% CI 0.94–1.03, p = 0.441), and smoking status (OR 0.89, 95% CI 0.41–1.95, p = 0.777) were not significantly associated with HRM completion.

In multivariable analysis of initial post-operative clinic follow-up, higher ADI rank (OR 0.97, 95% CI 0.94–1.00, p = 0.054) and older age (OR 1.02, 95% CI 1.00–1.05, p = 0.077) demonstrated borderline associations with follow-up completion but did not reach statistical significance. Travel distance (OR 1.00, 95% CI 0.99–1.00, p = 0.287), sex (OR 0.67, 95% CI 0.26–1.70, p = 0.395), BMI (OR 1.02, 95% CI 0.96–1.07, p = 0.596), and smoking status (OR 0.57, 95% CI 0.23–1.41, p = 0.220) were not significantly associated with follow-up completion (Table 2). Similarly In multivariable analysis of overall post-operative follow-up, higher ADI rank (OR 0.97, 95% CI 0.94–1.00, p = 0.051) and older age (OR 1.03, 95% CI 1.00–1.05, p = 0.064) demonstrated borderline associations with follow-up completion but did not reach statistical significance. Travel distance (OR 1.00, 95% CI 1.00–1.01, p = 0.377), sex (OR 0.66, 95% CI 0.25–1.69, p = 0.383), BMI (OR 1.01, 95% CI 0.96–1.07, p = 0.660), and smoking status (OR 0.55, 95% CI 0.22–1.38, p = 0.202) were not significantly associated with follow-up completion.

**Table 2:** Correlation of ADI and SES factors to pre-operative HRM completion and initial and overall post-operative clinic follow up

Outcome	Variable	Completed	Not completed	p-value
Pre-operative HRM	ADI (mean ± SD)	77.80 ± 17.80	85.73 ± 13.51	0.004*
	Age (years)	56.41 ± 17.35	57.32 ± 21.97	0.775
	Distance (miles)	101.63 ± 82.31	92.96 ± 52.47	0.335
	Sex (male %)	50.0%	48.6%	0.883
Initial post-operative follow-up	ADI (mean ± SD)	78.46 ± 17.79	86.00 ± 11.81	0.010*
	Age (years)	57.63 ± 18.53	49.83 ± 15.63	0.052
	Distance (miles)	96.97 ± 59.98	118.74 ± 147.30	0.198
	Sex (male %)	51.6%	37.5%	0.198
Overall post-operative follow-up	ADI (mean ± SD)	78.46 ± 17.79	86.00 ± 11.81	0.013*
	Age (years)	57.64 ± 18.48	49.43 ± 15.85	0.045
	Distance (miles)	101.23 ± 80.24	90.44 ± 50.87	0.532
	Sex (male %)	51.3%	39.1%	0.277

Values are presented as median (interquartile range) for ADI and mean ± standard deviation for continuous variables. P-values for ADI are based on Mann–Whitney U tests, while other continuous variables were compared using independent samples t-tests and categorical variables using chi-square tests. Analyses are restricted to patients with available ADI data (n = 181)

*Indicates statistical significance

## Discussion

**Fig. 3:**
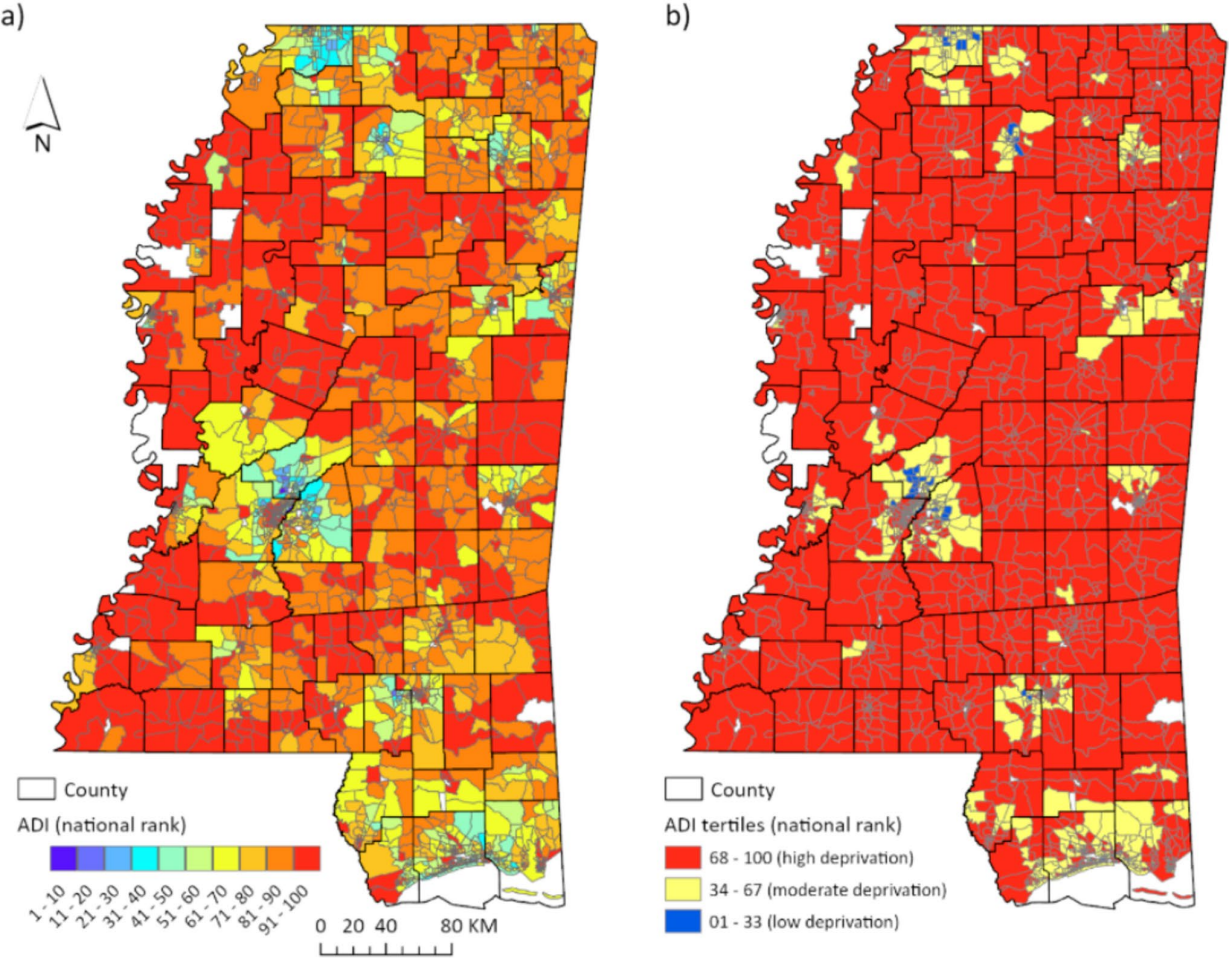
Mississippi national ADI in **a** declines and **b** tertiles

**Fig. 4:**
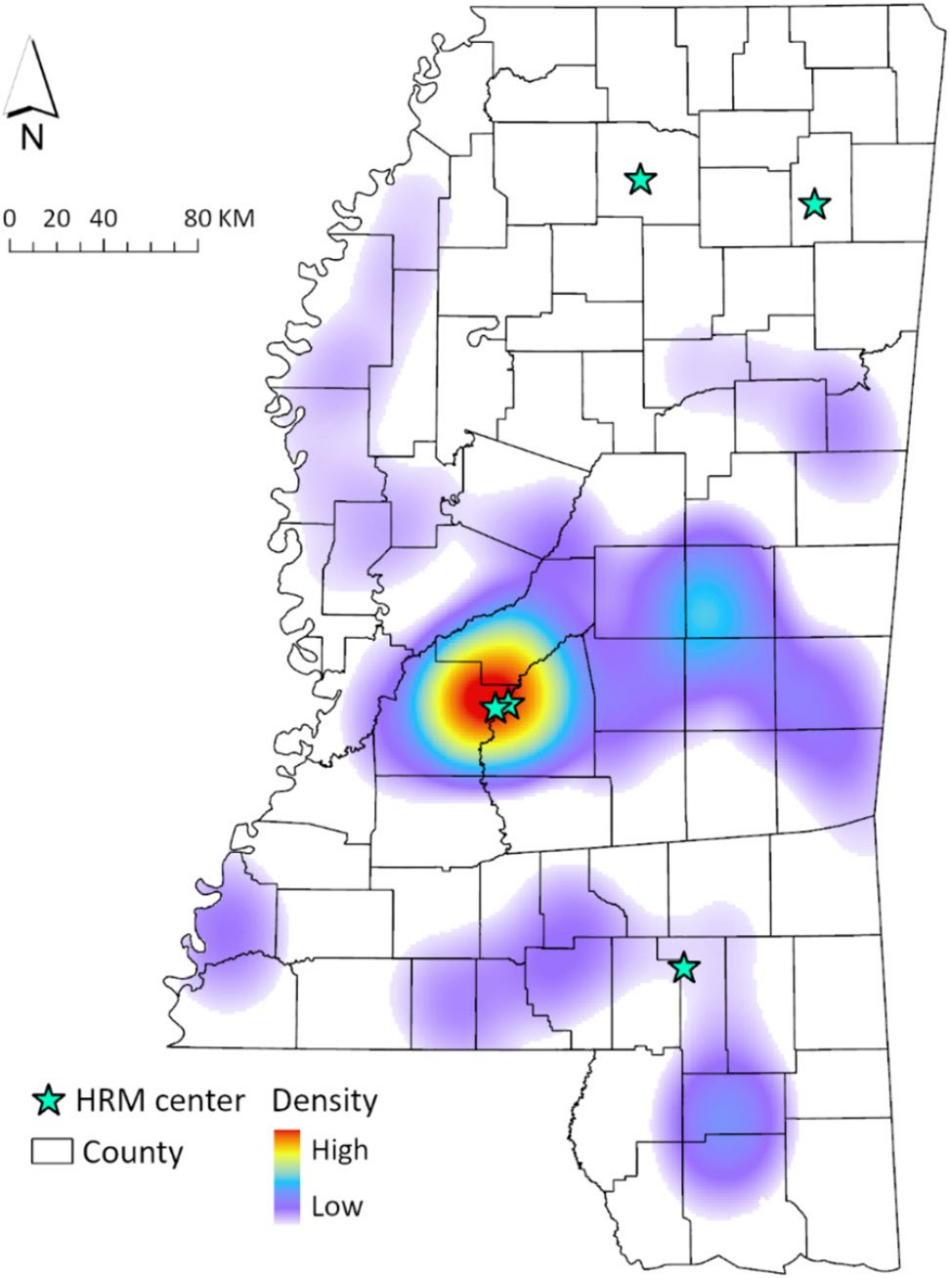
HRM non-compliance patient density and HRM centers in Mississippi

Our study demonstrates that neighborhood socioeconomic deprivation, measured by ADI, independently predicts failure to complete pre-operative HRM in patients undergoing POEM in Mississippi. Patients residing in more socioeconomically disadvantaged neighborhoods also demonstrated trends towards missing their post-operative follow up visits. These findings highlight the impact of neighborhood-level socioeconomic factors that can affect access to specialized diagnostic pre-operative testing as well as post-operative longitudinal care (Figs. 3, 4) [24].

Though ADI on follow up completion lost significance on multivariate analysis, it was still the most important barrier to HRM access even accounting for older age and longer drive times. These trends suggest that barriers to care extend beyond geographic location and may include other structural factors such as limited transportation availability, financial constraints, reduced health literacy, and limited access to reliable telephone or mobile services for additional pre-operative coordination. In this study, higher ADI was associated with HRM non-completion and may serve as a risk marker for identifying patients who require additional support.

The geographic context of our study further strengthens our findings. Mississippi has one of the highest proportions of rural residents in the United States with more than half of the state’s population residing in rural areas and the majority of counties designated as medically underserved or health professional shortage areas. The state’s disparities coupled with specialized esophageal motility and surgical endoscopic centers being concentrated in a limited number of urban areas augment the significance of these issues.

While there is limited prior data evaluating the role of socioeconomic disadvantage in regards to esophageal diseases and their diagnostic and post-procedural follow up, our study aligns with prior literature demonstrating that neighborhood deprivation is associated with disparities in surgical access and care, chronic disease outcomes, and healthcare utilization. From a clinical perspective, these results demonstrate that ADI may serve as a practical tool for risk stratification and identifying patients from high-deprivation areas at the time of referral to allow surgical and endoscopic centers to implement targeted interventions. ADI-based risk stratification can provide a framework for identifying patients at increased risk for barriers to specialized motility testing and longitudinal follow up with potential implementation strategies including targeted patient navigation services, early referral coordination, telehealth-based follow up visits, and transportation assistance. Implementing a comprehensive approach for high ADI patients with Achalasia undergoing POEM can address structural barriers to improve adherence to the recommended diagnostic evaluation and follow up.

Limitations of our study include limited power in our multivariable analysis given the small number of outcome events, particularly for post-operative follow-up, which raises the possibility of model overfitting and unstable estimates. Although covariates were selected based on clinical relevance, these findings should be interpreted with caution. Additionally, travel distance and travel time demonstrated substantial collinearity, reflecting their inherent relationship as measures of geographic access. To improve model stability, only travel distance was retained in the final models. Additional limitations included a retrospective single-center design and inclusion of patients who underwent workup at a tertiary center limiting generalizability to other institutions since patients with Achalasia who did not access specialty evaluation or completed diagnostic testing could have been excluded and underestimated the broader impact of socioeconomic deprivation on Achalasia care. Additional limitations include possible outcome misclassification, incomplete capture of outside HRM or outside follow up, residual confounding, overfitting due to small event counts, lack of individual-level socioeconomic variables, ecological fallacy from neighborhood-level ADI assignment, and lack of prospective validation.

Due to Mississippi’s higher poverty levels compared to national averages, ADI values based on national ranking were skewed toward higher deprivation categories. These findings can be applicable to other states with a rural-heavy population; however, it also limits generalizability to more urban health care systems with different referral structures and motility testing availability. A sensitivity analysis using state-level ADI rankings (ADI ranked within Mississippi) demonstrated that the association between socioeconomic deprivation and HRM completion remained significant (p = 0.016), supporting the robustness of the findings despite differences in ADI scaling.

Additionally, our study focused on neighborhood-level socioeconomic deprivation measured by the ADI rather than individual-level socioeconomic status measurements which may not fully capture each patient’s specific deprivation. We were also unable to directly measure potentially relevant barriers including digital access, caregiver responsibility, scheduling delays, out-of-pocket cost, insurance authorization, transportation reliability, health literacy, and patient understanding of HRM which can influence pre-operative HRM completion and follow up compliance.

In conclusion, our study demonstrates that access to high quality Achalasia care including pre-operative HRM and post-operative follow up is influenced by neighborhood-level socioeconomic conditions. Multidisciplinary efforts are needed to improve equity in specialized esophageal and motility care to account for social determinants of health especially in rural and medically underserved regions.

## Data Availability

De-identified data may be made available upon reasonable request in accordance with institutional and IRB guidelines.
